# Phytochemical profiles, antioxidant and antimicrobial activities of three *Potentilla* species

**DOI:** 10.1186/1472-6882-13-321

**Published:** 2013-11-19

**Authors:** Shan-Shan Wang, Dong-Mei Wang, Wen-Jun Pu, Deng-Wu Li

**Affiliations:** 1 College of Forestry, Northwest A & F University, Yangling, Shannxi 712100, China

**Keywords:** *Potentilla* spp, Phytochemicals, Antioxidant activity, Antimicrobial activity, RP-HPLC

## Abstract

**Background:**

Extracts from *Potentilla* species have been applied in traditional medicine and exhibit antioxidant, hypoglycemic, anti-inflammatory, antitumor and anti-ulcerogenic properties, but little has been known about the diversity of phytochemistry and pharmacology on this genus. This study investigated and compared the phytochemical profiles, antioxidant and antimicrobial activities of leaf extracts from three *Potentilla* species (*Potentilla fruticosa*, *Potentilla glabra* and *Potentilla parvifolia*) in order to discover new resources for lead structures and pharmaceutical products.

**Methods:**

Chemical composition and content of six phenolic compounds were evaluated and determined by RP-HPLC; Total phenolic and total flavonoid content were determined using Folin-Ciocalteau colourimetric method and sodium borohydride/chloranil-based method (SBC); Antioxidant activities were determined using DPPH, ABTS and FRAP assays; Antimicrobial properties were investigated by agar dilution and mycelial growth rate method.

**Results:**

The results showed hyperoside was the predominant phenolic compound in three *Potentilla* species by RP-HPLC assay, with the content of 8.86 (*P. fruticosa*), 2.56 (*P. glabra*) and 2.68 mg/g (*P. parvifolia*), respectively. The highest content of total identified phenolic compounds (hyperoside, (+)-catechin, caffeic acid, ferulic acid, rutin and ellagic acid) was observed in *P. parvifolia* (14.17 mg/g), follow by *P. fruticosa* (10.01 mg/g) and *P. glabra* (7.01 mg/g). *P. fruticosa* possessed the highest content of total phenolic (84.93 ± 0.50 mmol gallic acid equivalent/100 g) and total flavonoid (84.14 ± 0.03 mmol quercetin equivalent/100 g), which were in good correlation with its significant DPPH_IC50_ (16.87 μg/mL), ABTS (2763.48 μmol Trolox equivalent/g) and FRAP (1398.70 μmol Trolox equivalent/g) capacities. Furthermore, the effective methodology to distinguish the different species of *Potentilla* was also established by chromatographic fingerprint analysis for the first time. The results of antimicrobial activities showed *P. fruticosa* exhibited the strongest inhibition aganist Gram-positive bacteria, *Pseudomonas aeruginosa* and *Candida albicans* with MIC values of 0.78–6.25 mg/mL. *P. parvifolia* possessed antibacterial and antifungal activities against all the microorganisms tested, with EC_50_ and MIC values of 20.52–47.02 mg/mL and 0.78–50 mg/mL, respectively.

**Conclusions:**

These results indicated that leaf extracts from three *Potentilla* species could become useful supplement for pharmaceutical products as a new antioxidant and antimicrobial agents.

## Background

The genus *Potentilla* is a member of the family *Rosaceae*, which is mainly distributed in temperate, arctic and alpine zones of the Northern hemisphere. This genus has been known since ancient times for their decorative value and curative properties [1]. Extracts from the aerial and/or underground parts have been applied in traditional medicine and exhibit antioxidant, hypoglycemic, anti-inflammatory, antitumor and anti-ulcerogenic properties [2-6]. *Potentilla* extracts are preseumed to be safe and no toxic effects when applied to human [7,8]. In Tibet, *Potentilla anserina* root extracts have been applied for the treatment of certain viral infections as folk medicinal herbs [9]. Similarly the same or other *Potentilla* species have been used in traditional medicine of different cultures in Europe and Northern America [10]. Most of the biological effects of *Potentilla* species can be explained by the high amount of hydrolysable and condensed tannins, flavonoids and triterpenes present in all plant parts. Several of the polyphenols are identified as ellagic acid and flavonols glycosylated derivatives [11].

*P. fruticosa* is a species of hardy deciduous flowering shrub in the *Potentilla* genus of the family *Rosaceae*, native to the cool temperate and subarctic regions of the northern hemisphere, often growing at high altitudes in mountains [12]. In China, *P. fruticosa* also called “Jinlaomei” and “Gesanghua”, its altitude ranges from 400 to 5000 m [13]. Apart from its common gardening applications, it also has numerous medicinal virtues [14]. Extracts of *P. fruticosa* have been shown to possess relatively high concentrations of phenolic acids and flavonoids and powerful radical scavenging capacity [8,15]. The activity of some extracts was higher than that of the synthetic antioxidant BHT and of extracts isolated from sage (*Salvia officinalis*), which contains powerful antioxidants [5]. Moreover the leaves of *P. fruticosa* have applications as food additives and an ingredient in cosmetic products [16].

*P. glabra* is a small shrub species now occurring widely on the Himalayas and neighbor regions. It can reach the highest altitude of 5 000 m, indicating its high capability for cold endurance [17]. Differentiations between *P. glabra* and *P. fruticosa* are quite subtle, and can only be distinguished by the color of the petals (white versus yellow) [18]. *P. parvifolia* is another species belonged to the genus *Potentilla*. Widely distributed in Qinghai, Gansu, Inner Mongolia, Heilongjiang, Tibet and Sichuan in China, which can grow normally at an altitude of 1 500 to 4 500 m area. *P. glabra* and *P. parvifolia* are also referred to varietas of *P. fruticosa*[19], for the taxonomic status of the shrubby *Potentilla* is uncertain [12]. To our knowledge, the research of the phytochemicals and antioxidant properties for them are poorly investigated. As most of the biological activities of plant extracts can be explained by their high content of polyphenol [20,21], these two species may also become important sources for medicinal remedies as an alternative to chemical drugs, e.g. in antimicrobial therapy.

*Potentilla* species have been widely used for a long time in China as folk medicinal herbs and functional tea to treat diarrhoea, hepatitis, rheuma and scabies*,* but little has been known about the diversity of phytochemistry and pharmacology on this genus. Therefore, a comparison of the different *Potentilla* species would be desirable in order to discover the diversity of chemical constituents and quantities. In addition, the different types (or regions) of extracts and their phytochemical profiles should be investigated. This would be of high value in order to answer the question whether the phytochemical and pharmacological results for one *Potentilla* species can be transferred to another *Potentilla* species and also for one extracts within the same *Potentilla* species to another kind of extracts. Additionally, the genus *Potentilla* is abundant in polyphenolics which we detected might be introduced as chemotaxonomic markers of the different *Potentilla* species.

The objectives for this study were: (1) to determine the content of six phenolic compounds, total phenolics and total flavonoids in three selected *Potentilla* species; (2) To compare and screen the chemical composition of three *Potentilla* species by RP-HPLC assay; (3) to measure the antioxidant activity *in vitro* and (4) to determine the antimicrobial activity of three *Potentilla* species *in vitro*.

## Methods

### Plant material

Three samples were collected from different areas (Table 1) and identified by the Herbarium of the Northwest Sci-Tech University of Agriculture and Forestry. Collected plant materials were air-dried under shade at room temperature and were stored at −20°C and protected from light until further analysis.

**Table 1:** Species from different sources

**Species**	**Collected location**	**Part**
*P. fruticosa*	Huzhu Northern Mountain, Qinghai	Leaf
*P. glabra*	Helan Mountain, Ningxia	Leaf
*P. parvifolia*	Taibai Mountain, Shaanxi	Leaf

### Chemicals

Folin-Ciocalteu Reagent (Shanghai solarbio Bioscience & Technology Co., LTD, PR China); 1,1-diphenyl-2-picryl-hydrazyl (DPPH), 2,2-azino-bis(3-ethyl-benzothiazoline-6-sulphonic acid) di-ammonium salt (ABTS), 2, 4, 6-Tripyridyl-s-triazine (TPTZ), 6-Hydroxy-2,5,7,8-tetramethylchroman-2-carboxylic Acid (Trolox) (Sigma- Aldrich Co., St. Louis, USA); Gallic acid monohydrate (Kebang Bioscience & Technology Co., LTD, PR China); ethanol, acetone (Chengdu Kelong Chemical Co., Ltd, PR China); sodium borohydride (NaBH_4_), methanol, aluminum chloride, acetic acid, hydrochloric acid, vanillin, sodium dihydrogen phosphate, disodium hydrogen phosphate, sodium chloride, potassium chloride, potassium dihydrogen phosphate, sodium carbonate, sodium acetate, ferric trichloride hexahydrate (FeCl_3_ · 6H_2_O), methanol, potassium persulfate (Tianjin Bodi Chemical Co., Ltd, PR China); chloranil(Aladdin Industrial Co., Ltd, PR China); Tetrahydrofuran(THF) (Shenzhen Guanghua technology Co., Ltd, PR China); (+)-catechin, caffeic acid, ferulic acid, hyperoside, rutin, ellagic acid (Yuanye Industrial Co. Ltd, PR China). All other reagents and solvents used were of analytical grade. Deionized water (18MΩ cm) was used to prepare aqueous solutions.

### Preparation of the extracts

The air-dried and powdered sample (2 g) was extracted with 50 mL of 80% chilled acetone at 4°C for 1 h and then the mixture was filtered with vacuum pump. Extracts were transferred into a new test tube. The residues were extracted with additional 50 mL 80% chilled acetone twice using the same conditions. Extracts in the new test tube were evaporated to dryness at 45°C with rotary evaporators, the dry residue was dissolved in volumetric flask with methanol and stored at −20°C in the dark for further use. It can be diluted if necessary. All extractions were performed in triplicate.

### Determination of total phenolic content

The total phenolic content was determined using the Folin-Ciocalteau colourimetric method with some modification [22]. Samples were thawed and prepared at concentration of 0.2 mg/mL. Add 100 μL sample and 400 μL deionized water to glass culture tube. Add 100 μL Folin-Ciocalteu Reagent, mix well and let the samples stand for 6 minutes. Add 1 mL 7% sodium carbonate and 0.8 mL deionized water, mix and let stand for 90 minutes at room temperature and its absorbance was read at 517 nm with a spectrophotometer (Shimadzu UV-1800). The phenolic content was calculated as Gallic acid equivalent from the calibration curve of Gallic acid standard solutions (20–300 μg/mL) and was expressed as millimole Gallic acid equivalent per 100 g of dry weight (mmol equiv.GAE/100 g). Data were reported as mean ± SD for three replicates.

### Determination of total flavonoids content

The total flavonoid content was determined using the sodium borohydride/chloranil-based (SBC) assay [23]. Samples were thawed and prepared at concentration of 0.2 mg/mL. Then dried to dryness and reconstituted in 1 mL of THF/EtOH (1:1, v/v). Quercetin standards (0.1- 10.0 mM) were prepared fresh each day before use in 1 mL of THF/EtOH (1:1, v/v). Each test tube with 1 mL of sample solution or 1 mL of quercetin standard solution had 0.5 mL of 50 mM NaBH_4_ solution and 0.5 mL of 74.56 mM AlCl_3_ solution added. The tubes were shaken in a thermo shaker at room temperature for 30 min on setting 400. An additional 0.5 mL of 50.0 mM NaBH_4_ solution was added into each test tube with continuing shaking for another 30 min at room temperature. 2.0 mL of 0.8 M cold acetic acid solution (4°C) was added to each test tube, and the solutions were protected from the light for 15 min after a thorough mix. Then 1 mL of 20.0 mM chloranil was added into each tube. The tubes were placed in the dry bath incubator set at 95°C with shaking for 60 min in an orbital shaker. And then the reaction solutions were cooled using tap water, and the final volume was brought to 4 mL with methanol. 1 mL 16% vanillin methanol solution was added to each tube, mixing well. Then 2 mL of 12 M concentrated HCl was added to each tube and kept in dark at room temperature for 15 min after a thorough mix. Then the absorbance was measured at 490 nm using spectrophotometer. Data were reported as mean ± SD for three replicates. Total flavonoid content were expressed as millimole quercetin equivalents per 100 g of dry weight (mmol equiv. QUE/100 g).

### Reverse-phase HPLC analysis of six phenolic compounds

Stock solution was thawed and then analysed by RP-HPLC. The content of six phenolic compounds ((+)-catechin, caffeic acid, ferulic acid, hyperoside, rutin and ellagic acid) were quantified by using an Agilent Technologies 1260 series liquid chromatograph (RP-HPLC) coupled with a variable wavelength detector. The quantification was carried out on a SB-C_18_ reversed phase column (5 μm, 4.6*250 mm) at ambient temperature. The mobile phase consisted of water with 0.2% trifluoroacetic acid (solvent A) and methanol with 0.2% trifluoroacetic acid (solvent B). The following gradient elution program was run: 5% B (0 min), 20% B (0–10 min), 25% B (10–15 min), 25% B (15–20 min), 30% B (20–25 min), 30% B(25–35 min) , 35% B (35–40 min) , 45% B (40–50 min) , 100% B (50–60 min), 100% B (60–65 min). The mobile phase flow rate was kept at 0.8 ml/min. The injection volume was 20 μL, and the chromatogram was recorded at 320 nm and 360 nm. Analyses were performed in triplicate.

### *In vitro* antioxidant activity

#### DPPH free radical-scavenging assay

The scavenging effects of samples for DPPH radical were monitored according to the method of Yen [24]. Samples were diluted in ethanol at the following concentrations: 400, 200, 100, 50, 25, 12.5, 6.25 and 3.125 μg/mL. Briefly, a 2.0 mL of test sample was added to 2.0 mL of 0.1 mM DPPH methanolic solution. The mixture was mixed well and then left to stand in the dark for 30 min at room temperature, and its absorbance was read at 517 nm with a spectrophotometer against a blank. Trolox in the same concentrations was used as the positive control. All measurements were done in triplicate. DPPH free radical-scavenging activity was calculated according to the following equation:

$$\text{Inhibition}\;\mathrm{rate}\;\left(\%\right)=[1-(A_{i}-A_{j})/A_{0})]*100$$

Where A_0_ was the absorbance of methanol (2 ml) and DPPH (2 ml), A_i_ was the absorbance of the tested sample (2 ml sample and 2 ml DPPH), and A_j_ was the absorbance of the blank (2 ml sample and 2 ml methanol).

A lower absorbance of the reaction mixture indicated a higher DPPH radical-scavenging activity. IC_50_ values were the effective concentrations at which DPPH radicals were scavenged by 50%, and were obtained from linear regression analysis.

#### ABTS•^+^ radical cation scavenging assay

The method of decolourisation of free radical ABTS•^+^ was performed according to Re et al. with some modification [25]. The ABTS•^+^ was prepared by mixing an ABTS stock solution (7 mM in water) with 2.45 mM potassium persulfate. This mixture was allowed to stand for 12–16 h at room temperature in the dark until reaching a stable oxidative state. For each analysis, the ABTS•^+^ solution was diluted with pH 7.4 phosphate buffered saline (PBS) solution to an initial absorbance of 0.700 ± 0.021 at 734 nm. This solution was freshly prepared for each analysis. For the spectrophotometric assay, 100 μL extracts with a concentration (w/v) of 0.2 mg/mL was added to 3.9 mL of ABTS•^+^ solution and the absorbance was determined at 734 nm. Results were expressed in terms of micromoles trolox equivalent per g of dry weight (μmol eq. trolox/g). All determinations were carried out in triplicate.

#### Ferric reducing power (FRAP) assay

The method of FRAP assay used was a modified version of that reported by Benzie and Strain [26]. The method is based on the reduction of a colorless ferric complex 2, 4, 6-tripyridyl-s-triazine complex (Fe^3+^-tripyridyltriazine) to a blue-colored ferrous form (Fe^2+^-tripyridyltriazine) by the action of electron-donating antioxidants. The FRAP reagent included 300 mM acetate buffer (3.1 g C_2_H_3_NaO_2_ · 3H_2_O and 1.6 mL C_2_H_4_O_2_), 10 mM TPTZ solution in 40 mM HCl and 20 mM FeCl_3_ · 6H_2_O solution, with the ratio of 10:1:1(v/v). The extracts were prepared at a final concentration of 0.2 mg/mL. For each analysis, 400 μL of sample solutions was added to 3 mL of freshly prepared FRAP reagent. The reaction mixture was incubated for 30 min at 37°C in a water bath in the dark. Then, the absorbance of the samples was measured at 593 nm using the spectrophotometer. The trolox was used as the standard solution. The FRAP results were expressed in terms of micromoles trolox equivalent per g of dry weight (μmol eq. trolox/ g). All of the treatment groups were measured in triplicate.

### *In vitro* antimicrobial activity

#### Preparation of test solution

Each of the finely powdered samples (5 g) was extracted with 125 mL of 80% chilled acetone at 4°C for 1 h and then the mixture was filtered with vacuum. Extracts were transferred into a new test tube. The residues were extracted with one additional 125 mL 80% chilled acetone twice using the same conditions. Extracts in the new test tube were transferred into 50 mL volumetric flask with 80% acetone. It can be diluted if necessary. All extractions were performed in triplicate.

#### Microorganisms

Six bacterial strains and one fungal strain were procured from Microbial Culture Collection Center of Guangdong Institute of Microbiology, China. The strains used are *Staphylococcus aureus* (ATCC No. 6538), *Enterococcus faecalis* (ATCC No. 29212), *Bacillus subtilis* (ATCC No. 6633), *Escherichia coli* (ATCC No. 25922), *Klebsiella pneumoniae* (CMCC No. 46117), *Pseudomonas aeruginosa* (ATCC No. 27853) and *Candida albicans* (ATCC No. 10231).

Twenty test fungi species, *Alternaria alternata*, *Alternaria brassicae*, *Alternaria solani*, *Bipolaris sorokininan*, *Botrytis cinerrea*, *Cladosporium fulvum*, *Colletotrichum gloeosporioides*, *Verticillium dahliae*, *Dothiorella gregaria*, *Fusarium oxysporum*, *Glomerella cingnlata*, *Phacidiopycnis washingtonensis*, *Physalospora piricola*, *Piricularia oryzae*, *Rhizoctonia cerealis*, *Sclerotinia sclerotiorum*, *Thanatephorus cucumeris*, *Valsa mali*, *Venturia pyrina* and *Verticillium dahliae* were kindly provided by the College of Resources and Environment, Northwest A&F University, Yangling, China.

#### Minimum inhibitory concentration (MIC)

The minimal inhibitory concentration (MIC) of extracts for antimicrobial testing was determined by agar dilution method according to that approved by the National Committee for Clinical Laboratory Standards (NCCLS) with some modification [27]: a series of two fold dilutions of each extracts, ranging from 0.2 to 100 mg/ml, was prepared. Each of the test sterile petri dishes contained 9 ml of medium and 1 ml extracts of three *Potentilla* species, the solvent without extracts served as negative control and using benzylpenicillin as a positive control. The medium was inoculated with 3 μl aliquots of culture containing approximately 10^5^ CFU/ml of each organism. The bacterial strains were cultured on Mueller–Hinton agar (MHA) medium at 37°C for 24 h and fungal strains on potato dextrose agar (PDA) medium at 28°C for 48 h. Inhibition of organism growth in the plates containing test crude extracts was judged by comparison with growth in blank control plates. The MIC values were determined as the lowest concentration of extracts inhibiting visible growth of each organism on the agar plate.

#### Antifungal activities against plant pathogenic fungi

Mycelial growth rate method was conducted to evaluate antifungal activity of phytochemicals against plant pathogenic fungi [28,29]. The 20 test fungi were seeded on sterilized potato dextrose agar (PDA) media in petri dishes. Each of the test PDA sterile petri dishes contained 9 ml of molten medium and 1 ml extracts of three *Potentilla* species (with concentration of 50 mg/ml), the solvent without extracts served as negative control. The plates were then incubated at 28°C for 72 h. Zones of inhibition were measured and recorded. The percentage inhibition was calculated:

$$\left[\left({\text{Diameter}}_{\text{Control}}-{\text{Diameter}}_{\text{Test}}\right)/{\text{Diameter}}_{\text{Control}}\right]*100$$

By this procedure, five pathogenic fungi, with inhibition percentage over 50%, were chosen for further analysis.

Various extracts of three *Potentilla* species were tested separately for kinetic study and evaluation of antifungal activity of five selected fungi, including *Alternaria alternata*, *Alternaria brassicae*, *Glomerella cingnlata*, *Physalospora piricola* and *Venturia pyrina*. Each extracts solutions were serially diluted by the two-fold serial dilution method and added to PDA with final concentrations ranging from 6.25 to 100 mg /ml. Amphotericin disks were used as standard. And the concentration of the sample required for 50% inhibition rate (EC_50_) was calculated using linear regression analysis. The experiment was repeated thrice and the average values were calculated.

### Statistical analysis

All results were expressed as the mean ± standard deviation (SD). The significant difference was calculated by SPSS one-way ANOVA followed by Duncan’s test; values < 0.05 were considered to be significant.

## Results and discussion

### Total phenolic and flavonoid content

Total phenolic content of three leaf extracts are presented in Table 2. Significant difference was found among three leaf extracts (p < 0.05). The results showed that *P. fruticosa* had the highest total phenolic content (84.93 ± 0.50 mmol equiv. GAE/100 g), followed by *P. glabra* (69.21 ± 0.64 mmol equiv. GAE/100 g) and *P. parvifolia* (55.22 ± 0.75 mmol equiv. GAE/100 g). Tomczyk reported that *P. fruticosa* which obtained from the Hortus Botanicus Universitatis Masarykianae had high concentrations of total polyphenols (116.30 ± 3.90 mg GAE/g) [8]. In this work, the total phenolic content of *P. fruticosa* was higher than that had been reported by Tomczyk. Since all samples were collected from different places of origin, these differences may be due to diversity in biological (species, organ and developmental stage) and environmental factors [30].

**Table 2:** **Contents of total phenolic and flavonoids of leaf extracts of three *Potentilla *** **species**

**Species**	**Total phenolic content (mmol equiv. GAE/100 g)**	**Total flavonoid content (mmol equiv. QUE/100 g)**
*P. fruticosa*	84.93 ± 0.50^c^	84.14 ± 0.03^c^
*P. glabra*	69.21 ± 0.64^b^	72.76 ± 0.02^b^
*P. parvifolia*	55.22 ± 0.75^a^	41.87 ± 0.04^a^

mmol equiv. GAE/100 g: millimole gallic acid equivalent per 100 gram.

mmol equiv. QUE/100 g: millimole quercetin equivalent per 100 gram.

Each values represented in tables are means ± SD (N=3).

Values with different letters (a, b, c) within same column are significantly different (P<0.05).

Total flavonoids content of three *Potentilla* leaf extracts were measured (Table 2). *P. fruticosa* presented the highest flavonoid content (84.14 ± 0.03 mmol equiv. QUE/100 g), followed by *P. glabra* (72.76 ± 0.02 mmol equiv. QUE/100 g) and *P. parvifolia* (41.87 ± 0.04 mmol equiv. QUE/100 g). The flavonoid content of three leaf extracts were significantly difference from each other (p < 0.05). It is well known that both genetic and environmental factors play important roles on flavonoid composition and nutritional quality of plants. Therefore, these factors would be the key point for affecting the flavonoid content of three *Potentilla* species.

Tomczyk had found that *P. fruticosa* had high content of total flavonoid (7.0 ± 1.1 mg QUE /g) for the aerial parts [8]. This value was significantly lower than that reported in the present study. Due to we adopted the sodium borohydride/chloranil-based (SBC) assay to detect the total flavonoids, which can measure all types of flavonoids, including flavones, flavonols, flavonones, flavononols, isoflavonoids, flavanols, and anthocyanins[23]. Thereby the significant differences found between those values may arise from the use of two different analytical methods.

### HPLC analysis of three *Potentilla* species

#### Validation of the method

**Figure 1:**
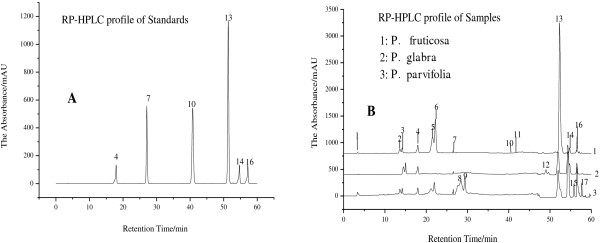
**Analytical HPLC chromatogram of reference substances (A) and phenolic compounds of leaf extracts of *P. fruticosa*, *P. glabra *** **and *P. parvifolia *** **(B): peak 4, (+)-catechin; peak 7, caffeic acid; peak 10, ferulic acid; peak 13, hyperoside; peak 14, rutin; peak 16, ellagic acid.**

Under the optimal conditions, six phenolic compounds in three leaf extracts were detected and well separated. The precision of the analytical method was determined by assaying six replicate of compounds 4, 7, 10, 13, 14 and 16 (Figure 1), the relative standard deviation (RSD) of the peak area were estimated to be 0.73–2.11% (N = 6). The repeatability of the method was detected by extracting one sample (*P. fruticosa*) for six times, while the area of the peaks were recorded, the RSD of the area varied from 0.65% to 3.28% (N = 6). A recovery experiment was performed to confirm the accuracy of the method by mixing quantified samples with standard compounds to appropriate amount. The average percentages of recovery of six phenolic compounds were at different levels and ranged from 94.81 ± 0.66% to 105.95 ± 2.44% and RSD varied from 0.36% to 2.88% (N = 6). The stability of compounds 4, 7, 10,13, 14 and 16 (Figure 1) in the sample solution were evaluated by determining their relative peak areas after storage at room temperature for 2, 4, 8, 16 and 24 h, respectively, the RSD of the relative peak areas were less than 3%. All results were summarized in Table 3 and demonstrated that the conditions for the analysis were repeatable and accurate.

**Table 3:** Method validation for the quantitative determination of six phenolic compounds using RP-HPLC

**Peak no.**	**Compounds**	**Regression equation**	**Precision experiment**		**Repeatability**		**Recovery experiment**	
			**Area of peak**	**RSD (%)**	**Area of peak**	**RSD (%)**	**Average recovery rate (%)**	**RSD (%)**
4	(+)-catechin	y = 2004.7x - 25.13(R^2^ = 0.9973)	83.12 ± 0.61	0.73	12.82 ± 0.22	1.74	101.54 ± 3.08	1.03
7	Caffeic acid	y = 146577x + 12.70(R^2^ = 0.9998)	323.05 ± 1.83	1.32	285.04 ± 2.94	2.94	102.66 ± 2.96	2.88
10	Ferulic acid	y = 141714x - 14.70(R^2^ = 0.9998)	416.81 ± 0.92	1.88	32.85 ± 0.87	1.32	105.95 ± 2.44	2.3
13	Hyperoside	y = 66172x + 37.96(R^2^ = 0.9986)	960.32 ± 5.22	2.11	5801.02 ± 17.51	2.66	105.72 ± 1.56	1.48
14	Rutin	y = 43663x + 30.06(R^2^ = 0.9964)	89.79 ± 0.65	0.76	239.68 ± 7.85	0.65	96.21 ± 0.34	0.36
16	Ellagic acid	y = 62654x - 82.44(R^2^ = 0.9999)	101.25 ± 1.43	0.93	181.42 ± 2.40	3.28	102.60 ± 1.20	1.17

Values were expressed in mean ± SD (N = 6).

#### Content of six phenolic compounds

Chromatograms of leaf extracts of three *Potentilla* species were analyzed by HPLC method. Seventeen peaks shown in the chromatograms were assigned as characteristic peaks. Based on comparison of the UV spectra and retention time of sample peaks to those of the standards, peaks 4, 7, 10, 13, 14 and 16 were identified as (+)-catechin, caffeic acid, ferulic acid, hyperoside, rutin and ellagic acid (Figure 1). Content of hyperoside was significantly high in extracts of three *Potentilla* species, especially in the extracts of *P. fruticosa* (8.86 ± 0.08 mg/g). Meanwhile, (+)-catechin, rutin and ellagic acid were the most abundant in the extracts of *P. parvifolia*, with the content of 2.53 ± 0.37, 4.11 ± 0.07 and 4.33 ± 0.09 mg/g, respectively. In addition, the content of caffeic acid (0.19 ± 0.00 to 0.51 ± 0.02 mg/g) and ferulic acid (0.01 ± 0.00 to 0.02 ± 0.00 mg/g) were relatively lower and not significantly different (p > 0.05) among three *Potentilla* leaf extracts. The content of total identified compounds varied significantly among three leaf extracts (p < 0.05). The highest was observed in the leaf extracts of *P. parvifolia* (14.17 mg/g), follow by *P. fruticosa* (10.01 mg/g) and *P. glabra* (7.01 mg/g). The results are summarized in Table 4.

**Table 4:** **Content of phenolic compounds of three *Potentilla *** **leaf extracts**

**Peak No.**	**Compounds**	**Rentention time(min)**	**Content (mg/g)**		
			***P. fruticosa***	***P. glabra***	***P. parvifolia***
4	(+)-catechin	17.96	0.60 ± 0.03^a^	0.54 ± 0.02^a^	2.53 ± 0.37^b^
7	Caffeic acid	26.29	0.19 ± 0.00^a^	0.33 ± 0.04^a^	0.51 ± 0.02^a^
10	Ferulic acid	41.34	0.02 ± 0.00^a^	0.02 ± 0.00^a^	0.01 ± 0.00^a^
13	Hyperoside	51.09	8.86 ± 0.08^c^	2.56 ± 0.11^a^	2.68 ± 0.05^b^
14	Rutin	53.77	0.05 ± 0.00^a^	2.56 ± 0.01^b^	4.11 ± 0.07^c^
16	Ellagic acid	56.61	0.29 ± 0.01^a^	1.00 ± 0.01^b^	4.33 ± 0.09^c^
Total identified	–	–	10.01^b^	7.01^a^	14.17^c^

Each values represented in tables are means ± SD (N=3).

Values with different letters (a, b, c) within same row are significantly different (P<0.05).

#### Chemical composition of three *Potentilla* species

The HPLC chromatograms of three *Potentilla* leaf extracts were shown in Figure 1b. Extracts of *P. parvifolia* possessed the richest peak numbers, followed by *P. glabra* and *P. fruticosa*. In detail, the peaks 1, 2, 3, 4 ((+)-catechin), 5, 6, 7 (caffeic acid), 10 (ferulic acid), 13 (hyperoside), 14 (rutin) and 16 (ellagic acid) were common peaks which were detected in all leaf extracts. Meanwhile, the content of peak 13 (hyperoside) was significantly higher in *P. fruticosa*. Peaks 4((+)-catechin), 14(rutin) and 16(ellagic acid) were the most abundant in *P. parvifolia*. However, peak 12 was only detected in *P. glabra*. Peaks 8, 9, 15, 17 were absent in *P. fruticosa* and *P. glabra*, but were found in *P. parvifolia.* In addition, peak 11 was typical in the extracts of *P. fruticosa* and *P. glabra*.

In total, this analysis of chromatograms provides a highly rational approach to the authentication and quality assessment of three *Potentilla* species through these characteristic peaks. And to our knowledge, this is the first report of the effective use of the methodology to distinguish the three different species of *Potentilla*. Furthermore, the considerable variation in the phytochemical composition of different extracts of *Potentilla* revealed by our research suggested that the technique reported here can also be used to determine relationships between phytochemical composition and therapeutic effects. Moreover, although this present study relates only to three *Potentilla* leaf extracts, the method has wider correlation for identifying species and quality assessment of other medicinal plants.

### *In vitro* antioxidant activity

#### DPPH free radical scavenging activity

**Figure 2:**
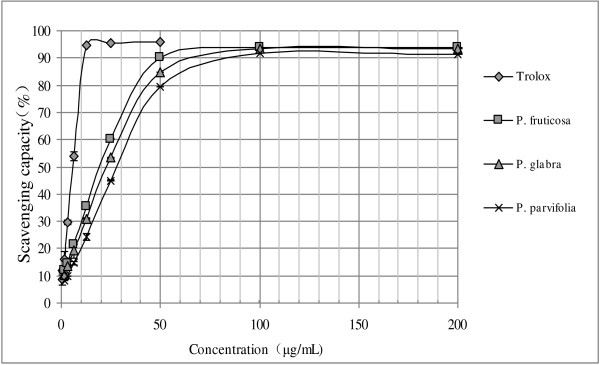
**DPPH scavenging activity (%) at various concentrations (μg/mL) of three *Potentilla *** **leaf extracts.**

The results of free radical scavenging properties of three *Potentilla* leaf extracts were showed in Figure 2 and Table 5. In DPPH assay, all extracts showed a notable radical scavenging activity in a dose-dependent manner within a certain range and were significantly different (p < 0.05). The highest antioxidant activity was obtained from the extracts of *P. fruticosa* with the lowest IC_50_ value of 16.87 ± 0.39 μg/mL, followed by *P. glabra* (19.37 ± 0.64 μg/mL) and *P. parvifolia* (23.87 ± 0.20 μg/mL).

**Table 5:** **Antioxidant activities of three *Potentilla *** **leaf extracts**

**Samples**	**DPPH**_**IC50 **_**(μg/mL)**	**ABTS (μmol Trolox/g)**	**FRAP (μmol Trolox/g)**
*P. fruticosa*	16.87 ± 0.39^c^	2763.48 ± 0.01^c^	1398.70 ± 8.29^c^
*P. glabra*	19.37 ± 0.64^b^	2192.16 ± 8.18^b^	1142.22 ± 0.80^a^
*P. parvifolia*	23.87 ± 0.20^a^	2140.22 ± 32.71^a^	1291.76 ± 0.01^b^
Trolox	4.28 ± 0.21^d^	−	−

Each values represented in tables are means ± SD (N=3).

Values with different letters (a, b, c, d) within same column are significantly different (P<0.05).

#### ABTS•^+^radical cation scavenging activity

ABTS activity was quantified in terms of percentage inhibition of the ABTS•^+^ radical cation by antioxidants in each sample. The ABTS values of the three samples were presented in Table 5. The results showed the same order of activity observed in the DPPH method. All extracts showed the capacity to neutralise the radical cation ABTS•^+^ and showed significant difference at P < 0.05. The highest activity was obtained from the *P. fruticosa* extracts with a value of 2763.48 ± 0.01 μmol equiv. Trolox/g, followed by *P. glabra* and *P. parvifolia* with values of 2192.16 ± 8.18 and 2140.22 ± 32.71 μmol equiv. Trolox/g, respectively.

#### Ferric reducing power (FRAP) assay

The FRAP assay evaluates the antioxidant properties of the extracts based on its reducing ability. The values obtained from three leaf extracts (Table 5) were significantly different (p < 0.05), but the order were inconsistent with the DPPH and ABTS assays. In this study, extracts of *P. fruticosa* still provided the highest antioxidant capacity with a FRAP value of 1398.70 ± 8.29 μmol equiv. Trolox/g, followed by *P. parvifolia* and *P. glabra* with values of 1291.76 ± 0.01 and 1142.22 ± 0.80 μmol equiv. Trolox/g, respectively.

Based on these results, it is possible to infer that leaf extracts of *P. fruticosa* not only presented the highest free radical scavenge capacity but also the strongest reducing capacity. It is well known that the antioxidant activity of a plant extracts largely depends on both the composition of the extracts and the test system, and cannot be fully evaluated by one single method due to the various mechanisms of antioxidant action, so more research need to be conducted.

#### Correlations between the total phenolic and flavonoids content and antioxidant activities

Table 6 showed the correlations (linear regression coefficients, R^2^) between the total phenolic content (TPC), total flavonoids content (TFC) and the three antioxidant assays results for three *Potentilla* leaf extracts. The DPPH method showed a good correlation with the TPC (R^2^ = 0.9617) and a very good correlation (R^2^ = 0.9906) with the TFC, indicating that most phenolic compounds extracted from three *Potentilla* leaf extracts were likely to contribute to the radical scavenging activity in this method.

**Table 6:** **Correlations values (R**^**2**^**) between the antioxidant activities and total phenolic content and total flavonoid content of three *Potentilla *** **leaf extracts**

**R**^**2**^	**TPC**	**TFC**
DPPH	0.9617	0.9906
ABTS	0.8376	0.5762
FRAP	0.1984	0.0278

ABTS results were well correlated (R^2^ = 0.8376) to the TPC but poorly with the TFC (R^2^ = 0.5762). It is known that only flavonoids of a certain structure and particularly hydroxyl position in the molecule determine antioxidant properties, in general these properties depend on the ability to donate hydrogen or electron to a free radical. This structure–activity dependency can be the explanation for the correlation decrease observed.

The FRAP method showed a very poor correlation with the TPC (R^2^ = 0.1984) and the TFC (R^2^ = 0.0278). It may happen that the leaf extracts of some compounds are good radical scavengers but poor reducing agents, thus leading to nonlinear results.

Accordingly, the variation of antioxidant capacity among them could be explained by their phenolic content and composition differences.

### Antibacterial and antifungal activities

#### Minimal inhibitory concentration

MIC values of three extracts are shown in Table 7. Six bacteria and one fungus were tested. The control (80% acetone) did not inhibit any of microorganisms tested. Among the plants tested, *P. fruticosa* showed the best activity against the Gram-negative bacteria (6.25 mg/ml) and Gram-negative bacteria *Pseudomonas aeruginosa* (6.25 mg/ml). Poor inhibitory activity was detected against another two Gram-negative bacteria (*Escherichia coli* and *Klebsiella pneumoniae*). The antimicrobial effects of *P. fruticosa* were previously studied, the difference is that Tomczyk found *P. fruticosa* was failed to inhibit the growth of Gram-(−) bacteria [31], but our results showed a higher antibacterial and antifungal activity than that of Tomczyk’s. This may be due to the different concentration of the single compounds and other phytochemicals in extracts show significant antimicrobial properties [32,33]. *P. glabra* also exhibited good MIC values against Gram-negative bacteria (6.25-12.5 mg/ml) and Gram-negative bacteria *Pseudomonas aeruginosa* (25 mg/ml), but failed to inhibit the growth of *Escherichia coli* and *Klebsiella pneumoniae*. On the other hand, *P. parvifolia* had inhibitory activity against both the Gram-negative bacteria and Gram-negative bacteria, and the MIC values were ranged from 12.5–50 mg/ml.

**Table 7:** **Minimal inhibitory concentration (MIC) values of three *Potentilla *** **leaf extracts**

**Micro-organisms**	**Tested materials (MIC mg/mL)**			
**Bacteria**	***P. fruticosa***	***P. glabra***	***P. parvifolia***	Standard*
*Staphylococcus aureus* (ATCC No. 6538)	6.25	6.25	12.5	0.002
*Enterococcus faecalis* (ATCC No. 29212)	6.25	12.5	50	0.03
*Bacillus subtilis* (ATCC No. 6633)	6.25	12.5	12.5	0.002
*Escherichia coli* (ATCC No. 25922)	>100	>100	50	>10
*Klebsiella pneumoniae* (CMCC No. 46117)	>100	>100	50	>10
*Pseudomonas aeruginosa* (ATCC No. 27853)	6.25	25	12.5	>10
**Fungi**				
*Candida albicans* (ATCC No. 10231)	0.78	1.56	0.78	>10

* Standard: Benzylpenicillin was used as standard antibiotic.

The results of the antifungal screening are presented in Table 7. Among the plants tested, *P. fruticosa* and *P. parvifolia* displayed the best activity against *Candida albicans* with MIC value of 0.78 mg/ml, followed by *P. parvifolia* (1.56 mg/ml).

#### Antifungal activities against plant pathogenic fungi

Leaf extracts of three *Potentilla* species had been studied for their preliminary antifungal activities against 20 plant pathogenic fungi (Table 8). By this procedure, five pathogenic fungi (*Alternaria alternata*, *Alternaria brassicae*, *Glomerella cingnlata*, *Physalospora piricola* and *Venturia pyrina*), with inhibition percentage over 50%, were chosen for further analysis. Leaf extracts of *Potentilla* showed various degrees of inhibition against the fungi tested (Table 9). *P. parvifolia* exhibited the strongest inhibition for *Alternaria alternata*, *Glomerella cingnlata*, *Physalospora piricola* and *Venturia pyrina* with the EC_50_ values of 42.56 ± 0.20, 25.71 ± 1.16, 47.02 ± 1.86 and 37.29 ± 0.49 mg/mL, it showed significant difference with *P. fruticosa* and *P. glabra* (P < 0.05). *P. fruticosa* showed the strongest antifungal activity on *Alternaria brassicae* (EC_50_ value =15.60 ± 1.88 mg/mL), there was no significant difference (P > 0.05) among the three leaf extracts for *Alternaria brassicae*. In total, *P. parvifolia* displayed higher antifungal activity against all the 5 fungi tested, followed by *P. fruticosa* and *P. glabra*.

**Table 8:** **Preliminary antifungal activity of three *Potentilla *** **leaf extracts (concentration used 50 mg/mL) against 20 plant pathogenic fungi**

**Pathogenic fungi**	**Inhibiting rate /%**		
	***P. fruticosa***	***P. glabra***	***P. parvifolia***
*Alternaria alternata*	50.91 ± 0.15^a^	50.91 ± 0.36^a^	56.36 ± 0.00^a^
*Alternaria brassicae*	60.87 ± 3.26^a^	63.04 ± 3.77^a^	56.52 ± 3.77^a^
*Alternaria solani*	49.18 ± 2.84^b^	47.54 ± 2.64^b^	39.34 ± 2.18^a^
*Bipolaris sorokininan*	49.09 ± 3.15^c^	25.45 ± 3.63^a^	36.36 ± 3.15^b^
*Botrytis cinerrea*	ND	ND	ND
*Cladosporium fulvum*	35.48 ± 2.79^a^	43.55 ± 2.79^b^	50.81 ± 1.40^c^
*Colletotrichum gloeosporioides*	54.55 ± 3.15^c^	41.82 ± 3.21^b^	32.73 ± 2.82^a^
*Verticillium dahliaee*	54.72 ± 2.83^b^	33.96 ± 3.27^a^	32.08 ± 0.01^a^
*Dothiorella gregaria*	28.00 ± 0.01^a^	32.00 ± 3.46^a^	28.00 ± 0.01^a^
*Fusarium oxysporum*	42.11 ± 3.26^b^	47.37 ± 0.04^b^	33.33 ± 3.04^a^
*Glomerella cingnlata*	61.76 ± 2.94^b^	52.94 ± 1.88^a^	52.94 ± 0.07^a^
*Phacidiopycnis washingtonensis*	46.55 ± 2.99^c^	37.93 ± 0.00^a^	43.10 ± 0.03^b^
*Physalospora piricola*	55.88 ± 2.94^a^	56.67 ± 2.38^a^	56.86 ± 3.40^a^
*Piricularia oryzae*	36.96 ± 3.77^b^	23.91 ± 3.17^a^	30.43 ± 3.76^ab^
*Rhizoctonia cerealis*	25.20 ± 3.02^a^	26.80 ± 2.08^a^	25.20 ± 2.50^a^
*Sclerotinia cerealis*	ND	7.27 ± 2.46^a^	11.36 ± 0.62^b^
*Thanatephorus cucumeris*	62.07 ± 1.98^c^	36.21 ± 2.99^a^	50.86 ± 2.59^b^
*Valsa mali*	43.64 ± 3.15^b^	32.73 ± 3.15^a^	45.45 ± 1.45^b^
*Venturia pyrina*	56.00 ± 3.46^b^	54.00 ± 1.73^a^	66.00 ± 3.46^c^
*Verticillium dahliae*	40.74 ± 3.21^c^	25.93 ± 3.21^a^	33.33 ± 0.01^b^

Each values represented in tables are means ± SD (N=3).

Values with different letters (a, b, c) within same row are significantly different (P<0.05).

ND: not determined at tested concentrations.

**Table 9:** **EC**_**50 **_**values of three *Potentilla *** **leaf extracts against five selected plant pathogenic fungi**

**Pathogenic fungi**	**EC**_**50 **_**value (mg/mL)**			
	***P. fruticosa***	***P. glabra***	***P. parvifolia***	Standard*
*Alternaria alternata*	46.04 ± 1.68^c^	47.79 ± 1.64^c^	37.29 ± 0.49^b^	0.43 ± 0.03^a^
*Alternaria brassicae*	15.60 ± 1.88^b^	20.34 ± 2.55^b^	20.52 ± 1.49^b^	0.06 ± 0.02^a^
*Glomerella cingnlata*	47.89 ± 0.30^c^	48.27 ± 0.07^c^	42.56 ± 0.20^b^	0.03 ± 0.01^a^
*Physalospora piricola*	54.98 ± 2.13^c^	63.28 ± 2.12^d^	47.02 ± 1.86^b^	0.48 ± 0.01^a^
*Venturia pyrina*	33.46 ± 1.39^c^	35.32 ± 0.46^c^	25.71 ± 1.16^b^	0.42 ± 0.01^a^

*Standard: Amphotericin was used as standard antibiotic.

Each values represented in tables are means ± SD (N=3).

Values with different letters (a, b, c, d) within same row are significantly different (P<0.05).

It is generally accepted that phenolic compounds present in plant extracts play an important role in their antimicrobial effects, and several studies claim that rutin, ellagic acid and hyperoside exhibit potent antimicrobial activity [25,33,34]. While the leaf extracts of *P. parvifolia* showed the highest content of analyzed phenolic compounds and largest peak numbers, this may be the reason for its high antifungal activity.

## Conclusions

Variation in phytochemical profiles, antioxidant and antimicrobial activities of three species of *Potentilla* are reported. The leaf extracts contributed 55.22–84.93 mmol equiv. GAE/100 g of total phenolic content and 41.87–84.14 mmol equiv. QUE/100 g of total flavonoid content. There were 11 common peaks which were detected in all leaf extracts by RP-HPLC assay. Moreover, six compounds ((+)-catechin, caffeic acid, ferulic acid, hyperoside, rutin and ellagic acid) were detected and quantified. Hyperoside was the predominant phenolic acid found in *Potentilla* species analysed (2.56–8.86 mg/g). It showed that significant differences in phytochemical content can exist among *Potentilla* species. Moreover, this analysis of chromatograms provides a highly rational approach to the authentication and quality assessment of three *Potentilla* species through these characteristic peaks. The antioxidant activity was assessed using the DPPH, ABTS•^+^ and FRAP assays. *P. fruticosa* possessed the highest antioxidant capacity with the DPPH_IC50_, ABTS and FRAP values of 16.87 μg/mL, 2763.48 and 1398.70 μmol Trolox equivalent/g, respectively. This work has shown that the phytochemicals present in *Potentilla* species have potent antioxidant activity and that the free radical scavenging capacity (DPPH and ABTS radical scavenging assays) in *Potentilla* species is positively correlated with total phenolic content. Additionally, *P. fruticosa* showed the best activity against the Gram-negative bacteria (6.25 mg/mL), Gram-negative bacteria *Pseudomonas aeruginosa* (6.25 mg/mL) and fungus *Candida albicans*(0.78 mg/mL), but failed to inhibit the growth of *Escherichia coli* and *Klebsiella pneumoniae. P. glabra* showed the same property as *P. fruticosa*. While *P. parvifolia* possessed inhibitory activity against all the selected microorganisms (0.78–50 mg/mL). *P. parvifolia* also showed the strongest inhibitions for the selected plant pathogenic fungi, this was suspected to be associated with its highest total content of six phenolic compounds (14.17 mg/g) as well as the richest peak numbers (15 peaks) or any other phytochemicals we had not detected. Despite ongoing scientific research on this species, this study constitutes the first attempt at comparing the phytochemical composition as well as the antioxidant and antimicrobial activities of three *Potentilla* leaf extracts. However, these compounds were only described for a limited number of *Potentilla* species. This genus and its quantification of phyto-constituents as well as pharmacological profile based on *in vitro*, *in vivo* studies and on clinical trials should be further investigated.

## Competing interests

The authors declare that they have no competing interests.

## Authors’ contributions

S-sW was responsible for the experiments and acquisition of data, analysis, drafting of the manuscript. D-mW was coordinator and designed all the assays. W-jP performed the investigation of phytochemical profiles experiments. D-wL made contribution to collection and preparation of herbal samples. All authors read and approved the final manuscript.

## Pre-publication history

The pre-publication history for this paper can be accessed here:

http://www.biomedcentral.com/1472-6882/13/321/prepub
